# The Chemical Probes Portal – 2024: update on this public resource to support best-practice selection and use of small molecules in biomedical research

**DOI:** 10.1093/nar/gkae1062

**Published:** 2024-11-18

**Authors:** Domenico Sanfelice, Albert A Antolin, Alisa Crisp, Yi Chen, Benjamin Bellenie, Paul E Brennan, Aled Edwards, Susanne Müller, Bissan Al-Lazikani, Paul Workman

**Affiliations:** Centre for Cancer Drug Discovery, Division of Cancer Therapeutics, The Institute of Cancer Research, London SM2 5NG, UK; Chemical Probes Portal, www.chemicalprobes.org; proCURE Department, Oncobell Program, Catalan Institute of Oncology (ICO) and Bellvitge Biomedical Research Institute (IDIBELL), Hospital Duran y Reynals Avinguda de la Gran Via, 199, L’Hospitalet del Llobregat, Barcelona, Catalonia, Spain; Chemical Probes Portal, www.chemicalprobes.org; Centre for Cancer Drug Discovery, Division of Cancer Therapeutics, The Institute of Cancer Research, London SM2 5NG, UK; Chemical Probes Portal, www.chemicalprobes.org; Centre for Cancer Drug Discovery, Division of Cancer Therapeutics, The Institute of Cancer Research, London SM2 5NG, UK; Chemical Probes Portal, www.chemicalprobes.org; Centre for Cancer Drug Discovery, Division of Cancer Therapeutics, The Institute of Cancer Research, London SM2 5NG, UK; Chemical Probes Portal, www.chemicalprobes.org; Centre for Medicines Discovery, Nuffield Department of Medicine, NDM Research Building, University of Oxford, Oxford OX3 7FZ, UK; Chemical Probes Portal, www.chemicalprobes.org; Structural Genomics Consortium, MaRS Centre, South Tower, 101 College St., Suite 700, Toronto, ON M5G 1L7, Canada; Chemical Probes Portal, www.chemicalprobes.org; Centre for Cancer Drug Discovery, Division of Cancer Therapeutics, The Institute of Cancer Research, London SM2 5NG, UK; Structural Genomics Consortium, Buchmann Institute for Molecular Life Sciences, Johann Wolfgang Goethe-University, Max-von-Laue-Straße 15, 60438 Frankfurt am Main, Germany; Chemical Probes Portal, www.chemicalprobes.org; Department of Genomic Medicine and the Institute for Data Science in Oncology, UT MD Anderson, University of Texas, MD Anderson Cancer Center, 1881 East Road, Houston, TX 77054, USA; Chemical Probes Portal, www.chemicalprobes.org; Centre for Cancer Drug Discovery, Division of Cancer Therapeutics, The Institute of Cancer Research, London SM2 5NG, UK; Chemical Probes Portal, www.chemicalprobes.org

## Abstract

The Chemical Probes Portal (www.chemicalprobes.org) is a free, public resource, based on expert-reviews, that supports the assessment, selection and use of small-molecule compounds that qualify as chemical probes. These high-quality reagents are essential for exploring the function of individual proteins in complex biological systems, such as cells and organisms, and for validating proteins as potential therapeutic targets. The use of reliable chemical probes accelerates protein annotation in basic biological studies and informs drug discovery. However, the use of low-quality compounds has historically led to erroneous conclusions in biomedical research, and experience shows that failure to follow best practice continues, an issue which the Portal aims to address. Here, we describe the latest updates to the Chemical Probes Portal in both content and functionality. The number of chemical probes and human protein targets covered has increased significantly, with improvements in the processes for obtaining expert reviews and user engagement. Moreover, new functionalities and enhanced tools have been introduced to better support biological researchers in selecting and using the best chemical probes for their studies.

## Graphical Abstract

**Graphical Abstract:**
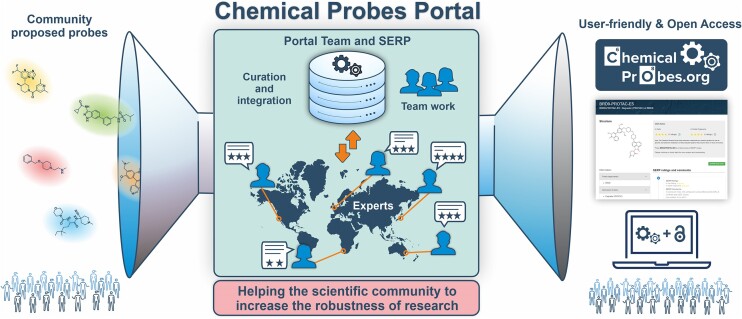

## Introduction

The Chemical Probes Portal (1) was established in 2015 as an internationally-led, web-based public resource, providing expert reviews and guidance on small-molecule probes to the biological research community (2). The best-practice use of high-quality chemical probes accelerates protein annotation in fundamental biological research and is an important part of the validation of therapeutic targets for drug discovery (2). Unfortunately, despite the considerable progress made and increasing use of small-molecule tools (3,4), it is clear that low-quality compounds are still commonly used and failure to follow best practice continues, leading to incorrect conclusions in the biomedical research literature (5).

Initially, the Chemical Probes Portal included a limited number of probes and covered a restricted range of protein families. With dedicated funding and community support, the number and diversity of probes on the Portal has been significantly enhanced and the coverage of protein families has increased, as well as the range of disease area relevance. In addition, the Portal now features greatly expanded content, a robust infrastructure, and improved user-friendly processes, available at our web site (www.chemicalprobes.org). Through the provision of easy-to-access advice on the selection of high-quality chemical probes acting selectively on the target proteins, as well as on the best-practice use of these reagents, the Portal aims to increase the reliability of research findings and thereby improve the robustness of biological and biomedical research.

The Chemical Probes Portal continues to be a non-profit resource, offering free, easy to access, expert assessments of chemical probes without registration; advice on probe selection and use; and recommendations on topics such as probe criteria, optimal concentration range, suitable control compounds to mitigate risk, and other use conditions, together with potential caveats. The Portal also provides curated bioactivity data for chemical probe assessment. In this update, we describe new features of the Portal, including improvements to probe submission, expert review, and governance processes. We highlight the expanded content for use by the scientific community and invite researchers to contribute to its ongoing development.

## Engagement with the research community and process

The Chemical Probes Portal is a community resource delivered through engagement of researchers in different roles. The core Portal team (www.chemicalprobes.org/people) is responsible for the development, maintenance, quality and coordination of all content and tools. While the Portal relies on the expertise of chemical biology and drug discovery researchers, it is aimed to be accessible to and support the broader biological scientific community at large, regardless of their expertise in chemistry or chemical biology.

Any user can recommend a new probe for review. Coordinated by the core team, probes are assigned to three members of our international Scientific Expert Review Panel (SERP; www.chemicalprobes.org/people#serp) who possess the specialist knowledge and expertise relevant to the chemical probe in question. The core team works with the SERP as part of a thorough editorial process and the reviews and ratings are published on the Portal website. The Chemical Probes Portal is overseen by a Board of Governors (www.chemicalprobes.org/people#board-of-governors) who support and guide the Portal team to ensure maximal benefit to the user community.

## Content development

**Figure 1.**
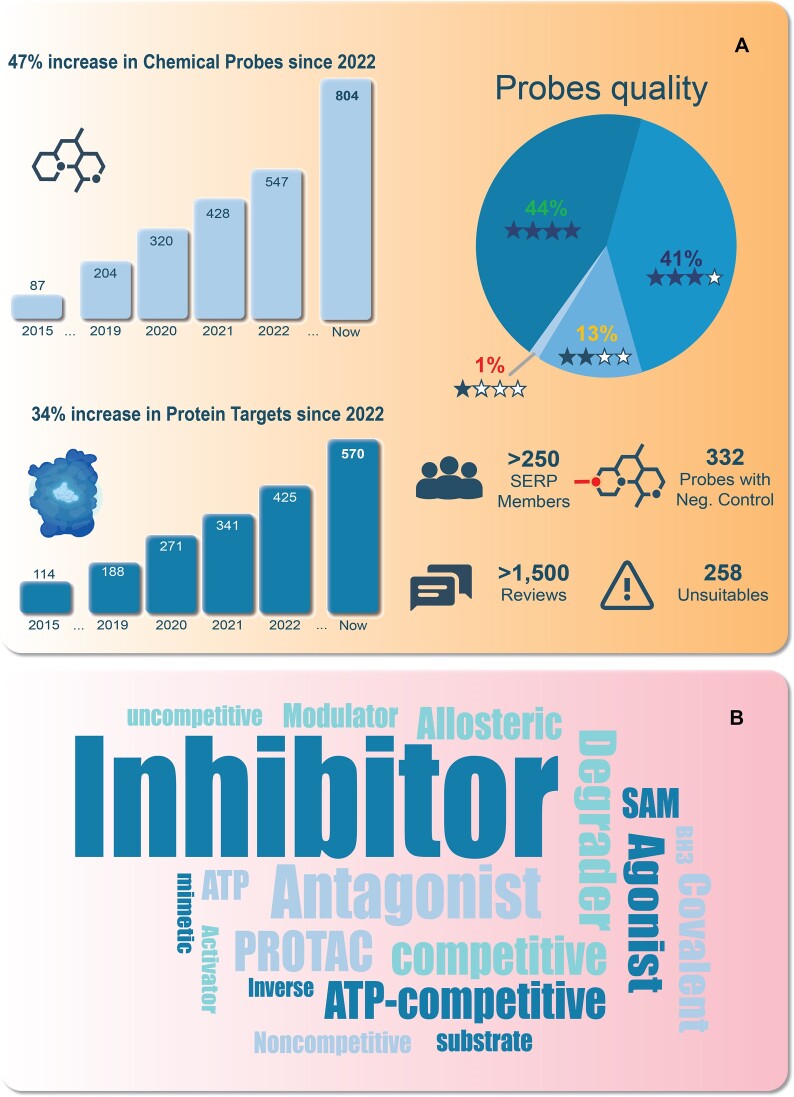
Some useful Portal metrics. (**A**) Total number of chemical probes and protein targets covered by the Chemical Probes Portal and their increase over time; distribution of star ratings for current chemical probes in the Portal, highlighting the overall high quality of the compounds in the database; number of SERP members, probe reviews, compounds with inactive negative controls available and unsuitables. (**B**) Word cloud highlighting the different modes of action of the chemical probes on the Portal.

Since the last update (1), the Chemical Probes Portal has seen substantial growth and improvements (Figure 1A). The database now includes 803 expert-annotated chemical probes, a significant increase of 47% from the previous count of 547 in 2022. Of these, about 82% of the compounds have been peer reviewed. The Portal team has established a rating system which is based on 4 rating levels, ranging from 1 to 4 stars (with 4 stars being the highest quality score) designed to help researchers assess the suitability of chemical probes for use in biological and biomedical research (www.chemicalprobes.org/info/rating-system). SERP members evaluate the data supporting each probe, considering important factors such as potency, selectivity, target engagement in cells, and mechanism of action, and then provide a star rating and associated review comments reflecting the probe's validity for use in cell culture systems *in vitro* and, if the probe has suitable properties (such as pharmacokinetic, target modulation and tolerability properties; see our guidelines www.chemicalprobes.org/info/guidelines-animals) a separate star rating score for use in animals, most commonly mice.

Probes with 3 or 4 stars are endorsed for use as specific high-quality modulators of their target, while those with fewer stars may lack sufficient validation to be recommended. The rating and associated review text ensures that researchers are informed about a probe's strengths, limitations, and optimal use contexts. Notably, 85% of the probes reviewed have been awarded either 3 or 4 stars for use in cells. Probes of this quality can be deployed with confidence for studies in cells and also, in many cases, in animals.

Most chemical probes (406) act as inhibitors for their primary target, but we are expanding and diversifying to include more probes with different molecular modes of action (www.chemicalprobes.org/menu/criteria-and-guidelines). For example, we now display 122 classical agonists/antagonists, 28 covalent binders and 51 degraders (6) (Figure 1B).

The substantial expansion of the number of chemical probes is accompanied by a 34% increase in the coverage of human protein targets, rising to 570 from 425 in 2022. We have also increased the diversity of targets and disease applications of the compounds, including those acting on protein targets relevant to neurodegenerative diseases like Alzheimer's and Parkinson's, as well as diabetes. This broader biomedical coverage is crucial for researchers working in areas outside the more heavily covered fields like oncology, and for which relatively few high-quality chemical probes are available to researchers.

For each probe reviewed, the Portal suggests a recommended concentration for use in cellular experiments, which is then reviewed by experts and made available to the community. As shown by a literature analysis published recently (5) by one of our Portal SERP members and colleagues, even high-quality chemical probes are often used inappropriately, with only 4% of publications employing the investigated chemical probes (which all featured on the Portal) within the recommended concentration range, together with the use of appropriate, available control compounds (see below). Utilization of inappropriate concentrations may often lead to erroneous conclusions by generating additional biological activity for which the probe is not annotated beyond its recommended concentration. Hence the recommended concentration range is an important feature of information provided on the Portal. As part of their specialist assessment, experienced reviewers are asked to comment on this crucial, yet underused, parameter, and this information is manually curated based on published data. We have a system in place that allows us to annotate this critical information, keeping it up to date, with the option to accommodate novel findings.

Where available, use of appropriately profiled control compounds is also very important but often not implemented (5). Hence, reviewers are also asked to comment on matched inactive (negative) and also orthogonal active probes from a different chemical class (chemotype). The Portal now features 332 compounds with an appropriate negative control – a structurally-matched compound with much less or no significant activity on the main target. Both negative and orthogonal active control compounds ideally need to be broadly profiled.

In addition, the Portal also highlights 258 compounds now designated as ‘Unsuitables’ – compounds which should not be used to interrogate the role of individual proteins because of poor selectivity and/or replacement by better quality probes (www.chemicalprobes.org/unsuitables).

Recognising their essential role in probe reviews, we have devoted significant efforts to expand and diversify our community of SERP members – with the number of experts now surpassing 250, representing both academia and industry. This growth in expertise has contributed to a 42% increase in the number of probe reviews since the last update in 2022, with the number on the Portal now totalling 1 522.

**Figure 2.**
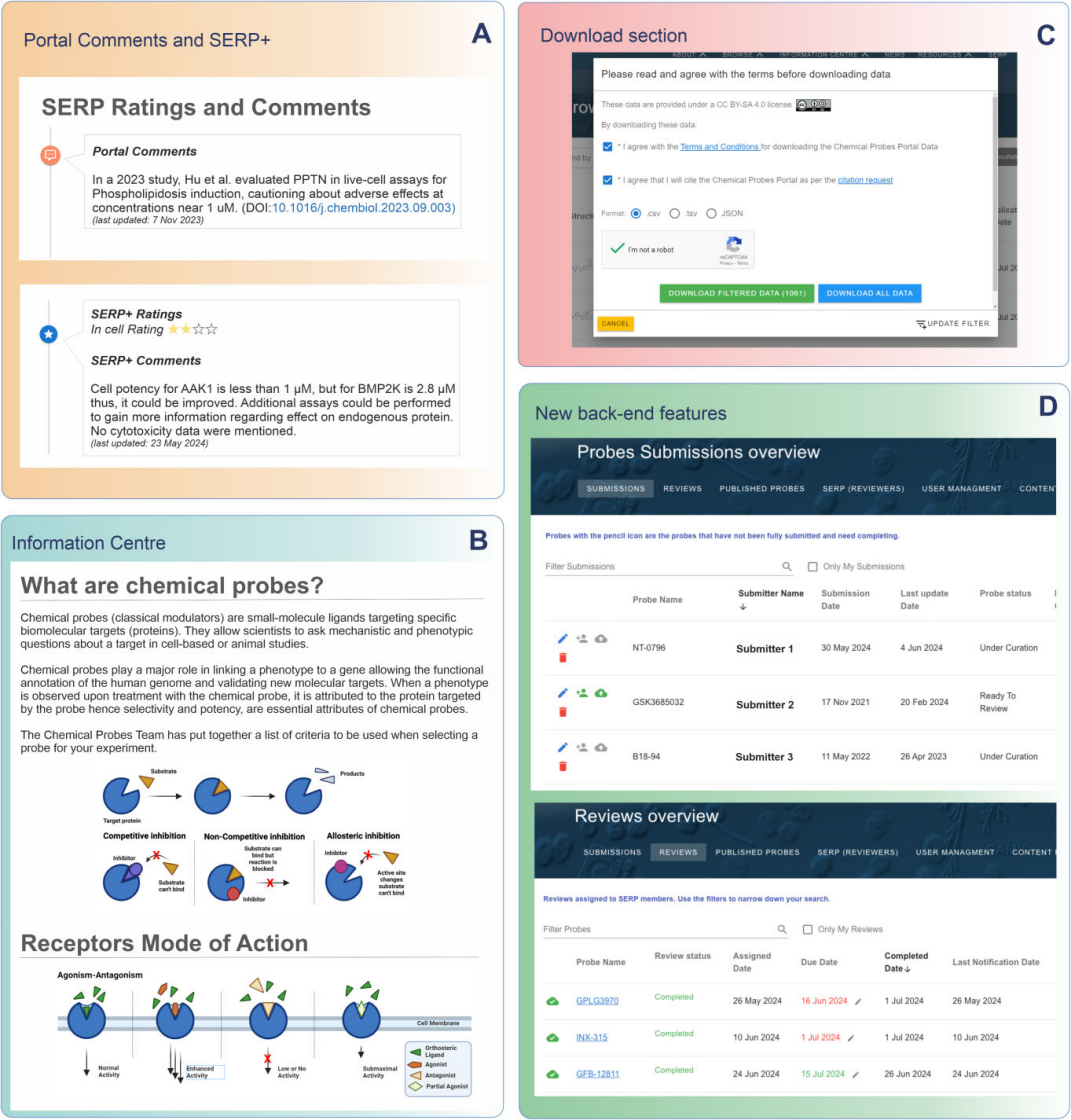
Some of the improvements now accessible for users, SERP members and administrators. (**A**) Portal comments keep users informed of the latest publications, and the review section features SERP + reviews generated from Chemical Probes Portal Hackathons. (**B**) The Information Centre now includes detailed information on chemical probes and their criteria, along with descriptions of molecular modes of action. (**C**) Users can now download data in various formats, making access to information easier and more flexible. (**D**) Administrators benefit from an updated back-end feature interface for sorting, evaluating, and publishing probe submissions; this new interface also facilitates the work of our SERP members.

As well as providing chemical probe reviews and associated information, such as probe criteria and guidelines for best-practice selection and use, education on the responsible, optimal utilization of chemical probes to ensure robust science is a key mission of the Chemical Probes Portal. In addition to lectures, webinars, journal publications, blogs, news features, and social media (posts on X and LinkedIn), we have initiated a new outreach activity, known as Chemical Probes Portal Hackathons (www.chemicalprobes.org/hackathons). The aim of these is to introduce the concept of chemical probes, how their characterisation should be critically reviewed, and the use of the Portal to younger generations and future leaders – in particular current PhD students – through supervised, active, live events where multiple chemical probes are reviewed. These events bring together experts and trainees for a day-long session focused on conducting as many thorough reviews of chemical probes as possible. Groups of students learn about chemical probes and work under the supervision of the experts to complete the assessments. Thus far, we have obtained ratings for over 100 compounds from these events, Thus Hackathons now regularly contribute to our reviews of probes. These curated reviews are published on the Chemical Probes Portal and are highlighted with the SERP + designation, indicating their origin from a Hackathon event (Figure 2A). The above hypertext link provides access to information on previous and future Hackathon events.

## Improvements for users

The information architecture of the website has been redesigned based on an in-depth user experience study, leading to a new structure for the menu and landing page, making it easier for users to navigate the site and find the information they need.

The Chemical Probes Portal has introduced several other new features to enhance the user experience. A new Information Centre (www.chemicalprobes.org/information-centre) has been established, for example providing detailed content pages on the various molecular modes of action of probes, eg including different types of receptor modulators (www.chemicalprobes.org/info/receptors-mode-of-action; Figure 2B). Additionally, updated criteria are provided for evaluating new modalities of chemical probes, including selective covalent binders and degraders (see https://www.chemicalprobes.org/menu/criteria-and-guidelines), thus helping to ensure that only the highest quality and most relevant probes are recommended.

A new feature allowing the Portal team to annotate and report new findings on published probes has been implemented, where relevant, on the individual probe pages. For example, this new feature has enabled the annotation of compounds that induce lipid accumulation, by drug-induced phospholipidosis (DIPL), which can be a confounder in many biological assays (7). In a recent publication, Portal staff authors and colleagues developed a robust method to predict and quantify DIPL (8). The relevant output of this publication is now annotated, where applicable, on the probe pages as ‘Portal Comments’. This facilitates the sharing of important data and the avoidance artefacts in cellular assays.

Based on an expert consultation, the category previously referred as ‘Historical Compounds’ has been renamed (see earlier) as ‘Unsuitables’, meaning not suitable for use as chemical probes to investigate any one particular protein in cells or organisms. Historical Compounds are deprecated small molecules that are not fit to be used as high-quality chemical probes. Although commonly historically relevant, they are often non-selective and/or not sufficiently potent compared with other available chemical probes. The term recognizes that many of these compounds were once valuable, for example if they were the first pathfinder compound available to study a protein target or target family, but are no longer suitable for this purpose and high-quality chemical probes better suited for this are recommended. We do acknowledge that some scientists may wish to select non-specific compounds for experiments precisely because they are non-specific, as these compounds may, for example, allow researchers to impact the activity of many proteins at the same time. Hence, our goal with this category of compounds is to indicate that although they can be useful in research they should not be used to interrogate the functionality of any specific individual cellular targets due to their poor or incomplete selectivity profile. Users have indicated that they find the Unsuitables list very helpful.

Another new feature on the home page is the display of current figures for the overall number of compounds, targets, and probe reviews, to provide users with a quick ‘at-a-glance’ numerical overview of the scale of key data currently hosted at the Chemical Probes Portal.

In addition, the download section (www.chemicalprobes.org/browse-probes) has been upgraded to include additional formats such as JSON, CSV and TSV, making it easier for users to access, download and utilize data in their preferred format (Figure 2C).

## Improvements for administrators

To streamline administrative tasks, several new backend improvements have been implemented. A comprehensive Content Management System (CMS) is now used to manage news, menu items, the Information Centre, personnel, sponsors and tutorials, providing administrators with better control and organizational possibilities. Requests from SERP members to conduct probe reviews are now sent to administrators via automated email, in addition to being stored in the database, ensuring timely notifications and easier tracking (Figure 2D).

An email checker feature has been added, allowing administrators to monitor sent and received emails more effectively. A new feature for content synchronization with the largest public cancer drug discovery resource, canSAR (9–14) (https://cansar.ai) has been introduced, enhancing data integration and integrity. The canSAR database standardizes and integrates more than 4 million small molecules with relevant biological data, including over 13 million bioactivity measurements, approximately 600 000 3D protein structures, and extensive biological and disease context data from molecular pathways to clinical trials. canSAR is updated weekly, allowing the rapid inclusion of new chemical data through its robust, recently improved chemistry pipeline (15). ChEMBL (16) (see www.ebi.ac.uk/chembl) constitutes the largest single source of compounds in canSAR, and in addition, canSAR also integrates chemical data from additional sources, including bespoke curated compounds and bioactivities (9–14). Live feeds from canSAR enable automatic updates of 3D protein complex information and other relevant data into the Portal.

Search Engine Optimization (SEO) has been improved to increase the Portal's visibility in Google searches. Enhanced control over the details and names of compounds has been implemented, ensuring accuracy and consistency. Additionally, new tools for managing reviewer activity, including reminders, decline tracking, and calendar integration, have been added to support the probe review process.

## Improvements for SERP members

The uniqueness, quality and value of the Portal content is highly dependent on the input from our SERP members. Therefore, several enhancements have been made to support their work. SERP members now have access to publications directly through the Portal, facilitating their research and reviewing activities. A new system for managing keywords has been introduced, allowing predefined classes, additional targets, and areas of expertise to be easily tracked (Figure 2D). This ensures that relevant SERP expertise is accurately categorized and utilized. SERP members can also proactively request a probe to review, in addition to reviewers that are assigned by the Portal team. Overall, the Portal has thus become considerably more user friendly for SERP members, which should in turn increase the efficiency and timeliness of the reviewing.

## Future developments

Looking ahead, the Chemical Probes Portal aims to implement several new features and expansions, with the main overall objectives being to continue to increase the number, diversity and proteome coverage of chemical probes featured; to provide information that helps the scientific community in the best-practice use of chemical probes; and to enhance effective outreach to non-expert communities.

To help further increase the content of the Portal, we will further automate the connectivity with major resources such as canSAR (https://cansar.ai/), the complementary statistically-based chemical probe assessment resource Probe Miner (https://probeminer.icr.ac.uk) and the Donated Chemical Probes initiative (www.sgc-ffm.uni-frankfurt.de). This will ensure that new key data are made available to users more easily and provides ready access to summarised information for SERP members to support their expert reviewing activity. Additional properties such as calculated probability for assay disruption properties such as phospholipidosis, aggregation, and solubility will be annotated, offering more comprehensive information on each compound.

Our ongoing collaboration with the Target 2035 initiative (www.target2035.net) will help to extend the Portal's reach, use and impact, including through webinars. Furthermore, we plan to enrich crosslinks to external chemical and target databases (e.g. PubChem, DrugBank, Therapeutic Target Database and Open Targets Platform), ensuring that users have seamless click-through access to a broader range of relevant resources.

We will continue to review and implement improvements to the chemical probes assessment process, focusing on obtaining ideally three timely reviews per probe and maintaining a dialogue for feedback from our SERP members, Board of Governors and the user community. This will also be helped by continuing to run the very successful Hackathon events, which is not only delivering sizeable batches of reviews, but in addition raises awareness and provides training for the next generation.

The updates and future plans described herein highlight our commitment to advancing the Chemical Probes Portal as a valuable, unique resource for the scientific community, ensuring the availability of information on the selection and use of high-quality chemical probes to the scientific community, and fostering collaborative efforts to accelerate robust biological and biomedical research discoveries.

## Data Availability

The Chemical Probes Portal is freely available at www.chemicalprobes.org.
